# Electrochemical Biosensor Designed to Distinguish Tetracyclines Derivatives by ssDNA Aptamer Labelled with Ferrocene

**DOI:** 10.3390/ijms232213785

**Published:** 2022-11-09

**Authors:** Kamila Malecka-Baturo, Apostolos Zaganiaris, Iwona Grabowska, Katarzyna Kurzątkowska-Adaszyńska

**Affiliations:** Department of Biosensors, Institute of Animal Reproduction and Food Research, Polish Academy of Sciences, 10-748 Olsztyn, Poland

**Keywords:** electrochemical biosensor, antibiotics, tetracycline, cow milk samples, aptamer, ferrocene

## Abstract

Controlling food safety and preventing the growing spread of antibiotics into food products have been challenging problems for the protection of human health. Hence, the development of easy-to-use, fast, and sensitive analytical methods for the detection of antibiotics in food products has become one of the priorities in the food industry. In this paper, an electrochemical platform based on the ssDNA aptamer for the selective detection of tetracycline has been proposed. The aptasensor is based on a thiolated aptamer, labelled with ferrocene, which has been covalently co-immobilized onto a gold electrode surface with 6-mercaptohexan-1-ol. The changes in the redox activity of ferrocene observed on the aptamer–antibiotics interactions have been the basis of analytical signal generation registered by square-wave voltammetry. Furthermore, the detection of tetracycline-spiked cow milk samples has been successfully demonstrated. The limits of detection (LODs) have been obtained of 0.16 nM and 0.20 nM in the buffer and spiked cow milk, respectively, which exceed the maximum residue level (225 nM) more than 1000 times. The proposed aptasensor offers high selectivity for tetracycline against other structurally related tetracycline derivatives. The developed biosensor characterized by simplicity, a low detection limit, and high reliability shows practical potential for the detection of tetracycline in animal-origin milk.

## 1. Introduction

Tetracyclines (TCs) are a group of antibiotics with broad-spectrum activity against Gram-positive and Gram-negative bacteria. The principal members of this group are tetracycline (TET), oxytetracycline (OTC), and doxycycline (DOX). These agents, owing to their beneficial antimicrobial properties and lack of serious side effects, have been widely used in human and animal treatments. Moreover, in some countries, animal feeds are supplemented with TCs in order to enhance the growth of animals [1]. Undoubtedly, improper administration, an insufficient withdrawal period after treatment, and general overuse of different antibiotics including TCs mainly in veterinary medicine have been responsible for the presence of these agents in milk and edible animal tissues [2,3]. In consequence, the development of worldwide antibiotic resistance has been observed [4,5]. Therefore, to protect the health of consumers, the maximum residue level (MRL) of pharmacologically active substances in foodstuffs of animal origin has been established (EUR-Lex—mi0026—EN—EUR-Lex (europa.eu)). The MRL for tetracycline in the milk of cattle is 100 µg/kg (CODEX ALIMENTARIUS).

Many analytical methods have been used to control antibiotic residues in different food samples, including (1) microbiological test kits [6], (2) chromatographic methods with various detection techniques including mass spectrometry [7], fluorescence detection [8], and ultra-violet detection [9], and (3) enzyme-linked immunosorbent assays [10] or enzyme-linked aptamer assays [11]. Traditional microbiological screening tests are simple and cheap but do not provide a clear indication of whether the substance is an antibiotic or not and what its concentration is [12]. Chromatographic techniques allow for performing qualitative and quantitative tests, as well as determining if there are metabolic products in the sample. However, the need for proper sample preparation, expensive apparatus, time-consuming analyses, and the requirement of highly trained technical personnel lead to important limitations. The ELISA tests characterized by high sensitivity and specificity for the detection of selected antimicrobial residues have become very popular [13]. Due to the time-consuming analyses consisting of several washing steps, the costs of analyses, and the lack of portability, the use of this technique in commercial laboratory practice is limited.

Biosensors combining two key elements, a recognition element and a transducer, have recently become high-throughput devices for fast, simple, selective, and ultrasensitive measurement of milk constituents [14,15,16]. The development of nanotechnology and advances in molecular biology have made biosensors cutting-edge devices for practical and real-time application and use in the field [17]. Among the different recognition elements, aptamers, single-stranded DNA, or RNA oligonucleotides, which can specifically bind to the target molecule, in this case, antibiotic molecules, with high affinity, selectivity, and sensitivity are of particular interest. Therefore, aptamers are promising alternatives as a recognition element in biosensors because they are more stable and sensitive than antibodies [18].

**Figure 1:**
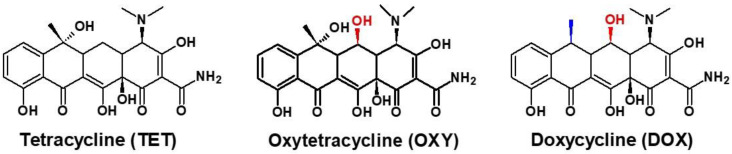
Chemical structures of tetracyclines.

Tetracycline derivatives are characterized by high structural similarity (Figure 1). However, a slight difference in the molecular structure affects the pharmacological properties, such as the elimination half-life (t_1/2_). t_1/2_ is longer for DOX (12–25 h) compared to TET and DOX, which have ranges of 6–11 h and 9.2 h, respectively [19]. Furthermore, the withdrawal periods for these antibiotics are different and depend on the veterinary medicinal products used. For tetracycline, the withdrawal period for cow’s milk is 96, but for oxytetracycline, it is 6 to 8 days. In contrast, doxycycline cannot be used to treat cows that produce milk for human consumption. Therefore, there is a need for a simple analytical method allowing for the discrimination of particular TCs. There are many electrochemical aptasensors for the detection of TCs in the literature. In most cases, the external redox marker or very complicated procedures of aptasensor construction have been applied. Two selected examples describe electrochemical aptasensors for the detection of tetracycline (TET) based on the biotinylated ssDNA aptamer immobilized on streptavidin-modified screen-printed gold electrodes [20]. Upon binding tetracycline to the aptamer, a decrease in the K_3_Fe(CN)_6_ peak current was observed using square-wave voltammetry (SWV). This system can detect TET with the minimum detection limit from sub-nanomolar to micromolar ranges. The authors did not test the potential application of aptasensor developed in food samples. In a further approach, a similar system has been tested for TET detection using electrochemical impedance spectroscopy (EIS) via an electrochemical probe of ferricyanide [21]. Here, a detection limit of 1.0 ng/mL and suitable recoveries of TET in samples of milk were obtained.

There is no doubt that the aptasensors reported so far show high efficiency in TCs detection. However, the use of very complicated procedures of aptasensor fabrication and the need for an external marker in the solution slightly reduce their practical significance. There is still a chance of finding systems with a simple configuration and straightforward operation. To our best knowledge, there is no example of electrochemical aptasensors based on ssDNA sequences labelled with ferrocene (Fc) for the detection of tetracycline. In this work, we present a report on an aptasensor based on a ferrocene–ssDNA conjugate for the electrochemical detection of tetracycline. We adopt an ssDNA aptamer sequence specific to TET selected by Niazi and co-workers [22]. The sensing of tetracycline has been registered by square-wave voltammetry of ferrocene attached to the ssDNA aptamer.

## 2. Results and Discussion

### 2.1. Electrochemical Characterisation of Gold Electrodes Modified with HS-Apt-Fc and MCH

**Scheme 1:**
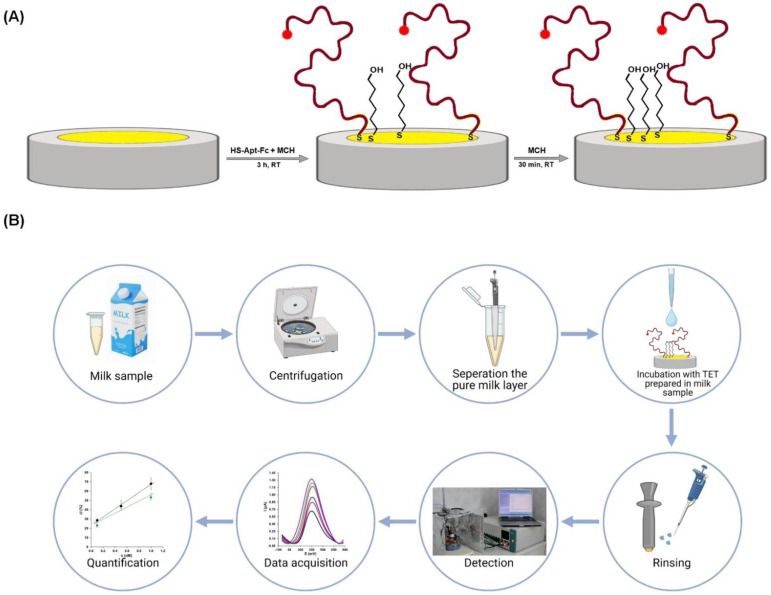
(**A**) Scheme of aptasensor preparation dedicated to TET detection. (**B**) Scheme of carrying out an experiment for the determination of TET using an electrochemical aptasensor.

The preparation of an electrochemical aptasensor for tetracycline detection is based on the two-step procedure of gold electrode modification with thiol compounds possessing affinity to the gold surface. According to the procedure presented in Scheme 1, in the first step, gold electrodes were modified in the mixture containing receptor molecules—5′-ferrocene-modified ssDNA aptamer (HS-Apt-Fc) selective for tetracycline and “diluent”—6-mercapto-1-hexanol (MCH), which is widely applied in the fabrication of electrochemical biosensors based on ssDNA sequences [23,24,25,26]. The second step, backfilling with MCH, was employed in order to inhibit biofouling [26,27].

**Figure 2:**
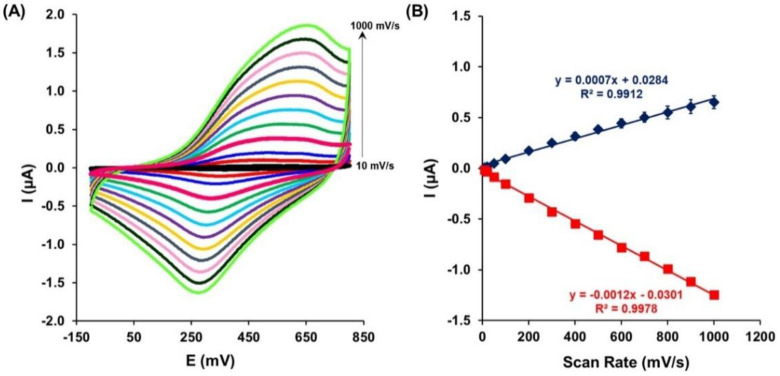
(**A**) Cyclic voltammograms recorded for the constructed aptasensor at different scan rates: 10, 20, 50, 100, 200, 300, 400, 500, 600, 700, 800, 900, and 1000 mV/s. (**B**) Linear relationship between the scan rates and redox peak currents observed for Fc. Potential measured vs. Ag/AgCl.

The receptor layer made of a gold electrode modified with HS-Apt-Fc and MCH has been electrochemically characterized. The presence of ferrocene attached to the ssDNA aptamer immobilized on the gold electrode surface was verified using cyclic voltammetry performed in the buffer. The oxidation (E_pa_) and reduction (E_pc_) peak of ferrocene attached to the aptamer were observed at the potential 480 ± 32 mV and 324 ± 8 mV, respectively, at a scan rate of 100 mV/s. To confirm the presence of the aptamer with ferrocene (Apt-Fc) on the gold electrode surface, the scan rate effect on the Fc oxidation and reduction peak current was investigated (Figure 2A). Furthermore, the relationship between the anodic and cathodic peak current positions vs. the scan rate (from 10 up to 1000 mV/s) was determined (Figure 2B). A linear dependence of the redox response upon the scan rate was observed, confirming the presence of Apt-Fc on the Au electrode surface and the adsorption-controlled process.

**Figure 3:**
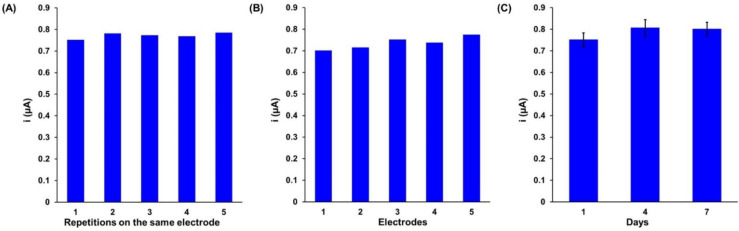
Results of (**A**) repeatability, (**B**) reproducibility, and (**C**) stability studies obtained for the gold electrodes modified with Au-S-Apt-Fc/MCH.

The aptasensor based on the ferrocene-labeled ssDNA aptamer was also characterized by determining parameters such as reproducibility, repeatability, and stability over time (Figure 3 and Table 1). Adequate reproducibility and repeatability of the aptasensor proposed were observed, proven by relative standard deviation (RDS) values of 1.7% and 3.9%, respectively (Figure 3A,B and Table 1).

Long-term stability is an important parameter in practical applications of the aptasensor. As shown in Figure 3C and Table 1, the signal changes after 7 days are minimal, and this indicates that the aptasensor has maintained its detection stability compared with the first day. Our results show that the ferrocene peak current increased by approximately 7% of its initial value. The obtained stability and reproducibility of the constructed aptasensor intensify the utility of the aptasensor.

### 2.2. Analytical Performance of the Aptasensor

In order to obtain optimal analytical performance of the presented aptasensor, two key experimental parameters, which could have a significant impact on the electrochemical signal of the aptasensor, have been optimized. Namely, the effect of incubation time and the TET concentration range on the aptasensor response has been investigated by square-wave voltammetry. Based on the obtained experimental results, 1 h was chosen as the optimal TET incubation time, while the concentrations range of 0.1, 0.5, 1.0, 5.0, and 10.0 nM was used to study the Apt-Fc–TET interactions.

**Figure 4:**
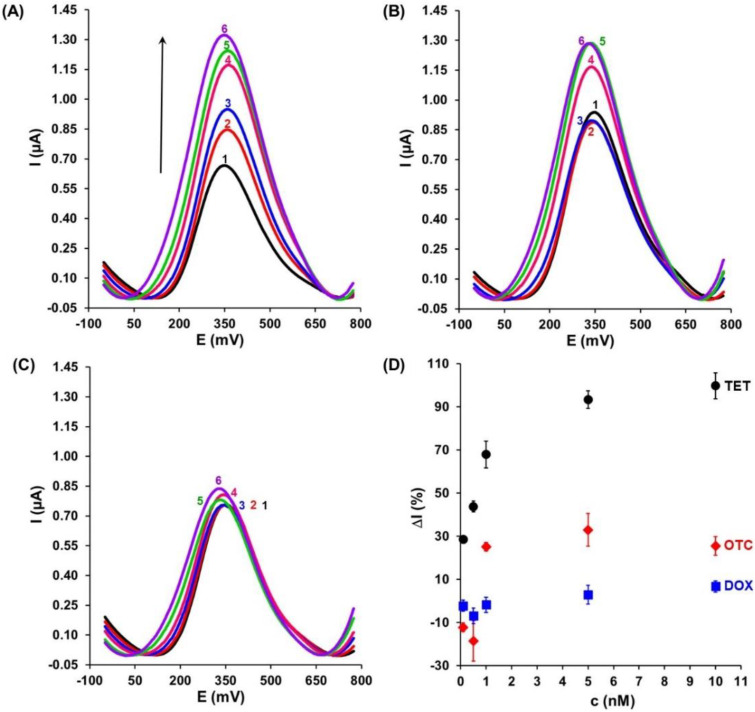
Example of square-wave voltammograms registered for gold electrodes modified with Au-S-Apt-Fc/MCH upon interaction with (**A**) TET, (**B**) OTC, and (**C**) DOX. **Black** curve (**1**) before and next curves after 1 h interaction with antibiotics at particular concentrations: 0.1 nM (**red**, **2**), 0.5 nM (**blue**, **3**), 1.0 nM (**pink**, **4**), 5.0 nM (**green**, **5**), and 10.0 nM (**violet**, **6**). (**D**) Relative intensity of current (ΔI) vs. concentration of (●) TET, (♦) OTC, and (■) DOX for gold electrodes modified with Au-S-Apt-Fc/MCH.

Based on the optimized measurement conditions, the SWV responses of the aptasensor at different concentrations of TET (0.1, 0.5, 1.0, 5.0, and 10.0 nM) have been registered. As presented in Figure 4A, the electrochemical signals gradually increased with increasing TET concentrations. The binding of 10nM TET to the Apt-Fc generated a 99.8 ± 6.0% increase in the Fc peak current (Table 2). A linear range was obtained from 0.1 to 1.0 nM.

The selectivity of the aptasensor is an important factor affecting the analysis performance of the aptasensor. To estimate the selectivity of this device, two target molecules structurally similar to TET (OTC and DOX) were tested separately under the same conditions. As shown in Figure 4B and C, in the presence of 10 nM OTC and DOX, an increase in the signal of 25.6 ± 4.3% and 6.7 ± 2.6% was recorded, respectively (Table 2). Compared to the TET response, these responses were significantly weaker. In this case, no linear response was observed. This could be explained by the high specific recognition ability of aptamer toward TET and also suggested that this tool possesses excellent selectivity for its target antibiotics. Figure 4D displays a relationship between the variation value of the electrochemical signal relative to the blank (ΔI) and the concentration of TET, OTC, and DOX, respectively, from 0.1 to 10 nM based on the data from Table 2.

**Scheme 2:**
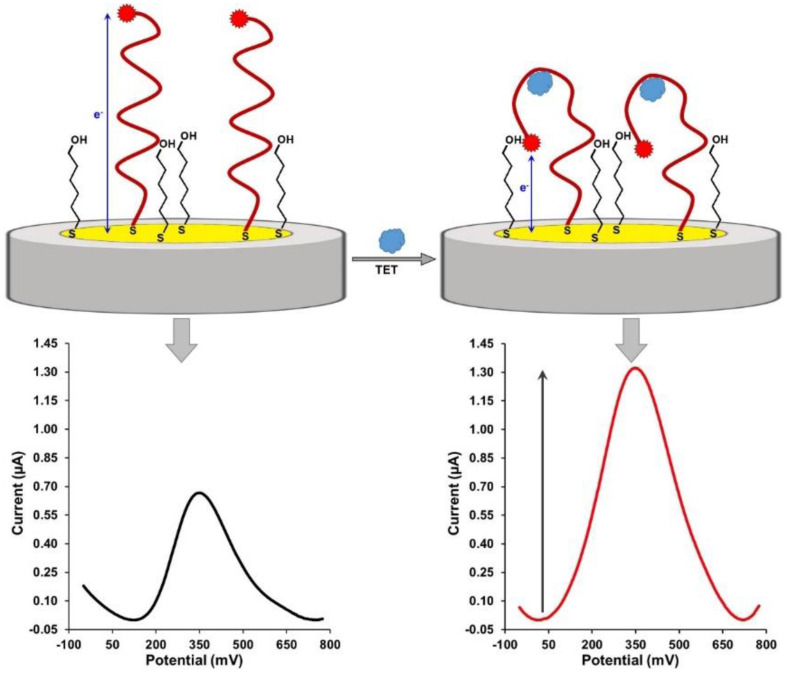
General scheme of ferrocene-based electrochemical aptasensors “signal-on” working principle.

The presented aptasensor worked according to the “signal-on” principle, generating an increase in the electrochemical signal after the introduction of the analyte molecule into the system [24,28]. Ferrocene-tagged aptamer molecules bind to TET targets via shape recognition. This means that upon the recognition process, aptamer molecules change their structure in such a way that the ferrocene moiety has a better chance of exchanging electrons with the electrode surface, leading to the increased peak current. Scheme 2 illustrates the general scheme of the “signal-on” mechanism of analytical signal generation.

### 2.3. Application of the Aptasensor

**Figure 5:**
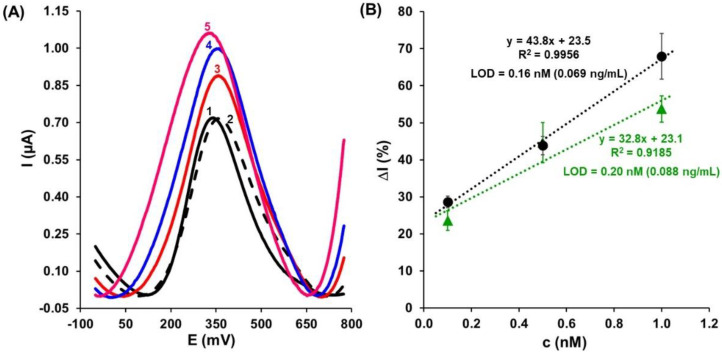
(**A**) SWV responses registered for different concentrations of TET prepared in pre-treated cow milk: 0.1nM (**red**, **3**), 0.5nM (**blue**, **4**), and 1.0nM (**pink**, **5**). **Black curve—**registered in buffer (**1**), **dashed curve**—registered in the cow milk matrix (**2**), and next curves after 1 h interaction with TET. (**B**) The calibration curves of aptasensor recorded for TET in (●) buffer solution and (▲) spiked cow milk with the concentration range of 0.1–1.0 nM (n = 4).

The good analytical parameters such as the linear responses in the range of 0.1 to 1.0 nM towards TET present in the buffer solutions, as well as the negligible responses to OTC and DOX (Figure 4D), suggest that the aptasensor under study could be successfully applied for the detection of TET in milk samples. Hence, the practical application of the developed aptasensor was evaluated for real cow-milk samples. The representative square-wave voltammograms are presented in Figure 5A. In the first step, the cow milk matrix influence on the prepared aptasensor (Au-S-Apt-Fc/MCH) was investigated. The black continuous line and black dashed line were recorded for the buffer and pre-treated cow milk, respectively (Figure 5A). Overall, the presence of the cow milk matrix generates no significant change in the square-wave voltammograms. In the next stage, the sensitivity of the developed aptasensor to TET present in the cow milk matrix, spiked with TET at various concentrations, was investigated. The tetracycline concentrations within the linear sensor response range recorded in the buffer were used, i.e., 0.1, 0.5, and 1.0 nM. The results were very similar to those recorded in the presence of buffer solutions alone. For increasing TET concentrations, increased peak currents of Fc were observed: 23.6 ± 2.6%, 44.6 ± 5.4%, and 53.7 ± 3.5%, respectively (Table 2).

Figure 5B illustrates the relationships between the variation value of the electrochemical signal relative to the blank buffer or cow milk matrix (ΔI, %) and the concentration of TET in the 0.1 to 1.0 nM range registered in the buffer (●) and spiked milk (▲). The limits of detection (LODs) were estimated to be 0.16 nM (0.069 ng/mL) in the buffer and 0.2 nM (0.088 ng/mL) in the cow milk matrix, using the following equation:

(1) $$LOD=\frac{3.3S}{a}$$

where *S* is the standard deviation for the blank solution and *a* is the slope of the calibration curve. Based on the experimental data, it can be interpreted that responses of the aptasensor registered in the buffer and spiked cow milk are comparable in terms of LOD. These results proved that the developed aptasensor has been successfully applied for the detection of TET in cow milk matrix samples.

**Figure 6:**
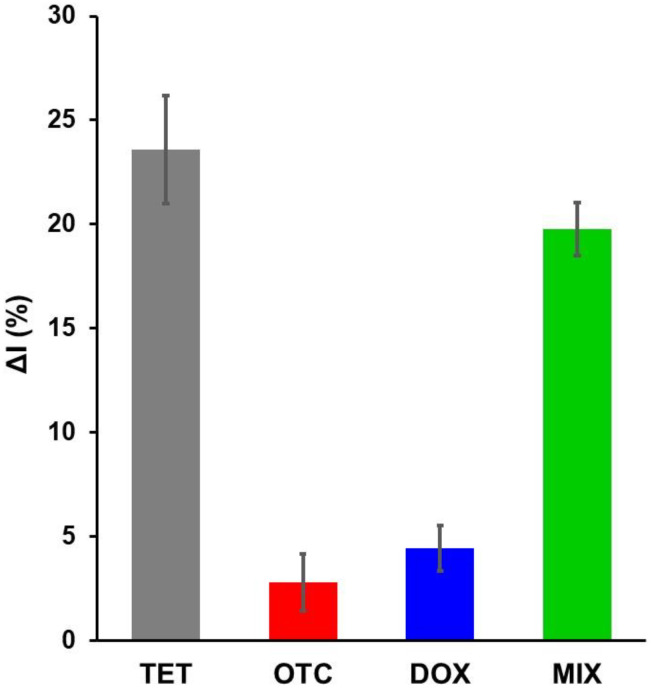
Selectivity of the prepared aptasensor in cow milk spiked with TET, DOX, OTC, and mixture of antibiotics: TET, DOX, and OTC with the same concentration of 0.1 nM (n = 4).

The presence of the cow milk matrix has a negligible influence on the selectivity of the proposed aptasensor. In Figure 6 and Table 2, relative changes of the Fc redox current (ΔI, %) are presented, caused by 0.1 nM concentrations of TET, OTC, and DOX present separately in the cow milk matrix and the mixture of all three antibiotics together. The response toward OTC and DOX of Apt-Fc, measured as the ΔI of Fc redox current, was ca. 3 times stronger in the cow milk matrix (2.8 ± 1.4% and 4.4 ± 1.1%, respectively), than in the buffer (−12.2 ± 2.2% and −2.2 ± 2.6%, respectively). The 0.1nM of TET caused a 28.7 ± 1.6% and 23.6 ± 2.6% increase in the Fc peak current recorded in the presence of the buffer solution and the cow milk matrix. Moreover, the ΔI registered for the mixed solution containing TET, OTC, and DOX is equal to 19.8 ± 1.3% and is comparable to the pure TET solution. The results suggest that this aptasensor shows exceptional selectivity for the detection of TET, which is attributed to the specific interaction between the aptamer and the TET. Despite the increase in the strength of the Apt-Fc response toward OTC and DOX, the selectivity of the present sensor is still satisfactory.

Many different methods have been employed for the detection and identification of TET in milk samples [29,30]. In Table 3, different methods for tetracycline detection in cow milk samples are presented. For example, the test-lux microbiological test and snap immunoassay were found to be reliable in detecting tetracycline residues in raw milk at >6.3 ng/mL. However, several tests such as the Charm HVS receptor assay and Delvotest SP microbial inhibition test do not fulfill the EU MRL required sensitivity criteria for TET. Generally, microbiological inhibition tests are time-consuming and not specific to certain groups of antibiotics. On the other hand, immunoassays and receptor-based assays are fast but sometimes not sensitive enough, more expensive, or need radioactive labels. Moreover, the incidence of false positives was rather high in many cases, mainly because of the presence of fatty acid molecules, proteins, somatic cells, bacteria, and natural antimicrobial compounds in raw milk samples. So, the applicability of these tests has to be considered with great caution [29,30].

In order to fulfill the criteria for simple, inexpensive, reliable, and sensitive tests for the detection of TET, various electrochemical aptasensors architectures have been reported. The use of nanomaterials [31,32], for example, the combination of reduced graphene oxide (RGO) and gold nanoparticles (AuNPs) as a nanocomposite, has undoubtedly caused the amplification of signals toward TET detection. However, the disadvantage of this kind of system is the necessity of using an external redox marker (Fe(CN)_6_^3−/4−^) and the need to add extra steps to its preparation process, making it more complex. There are many examples of electrochemical aptasensors that are based on very complicated procedures regarding their preparation. In an example approach, the electrochemical determination of TET was gained by a coupled triple-helix aptamer probe (TAP) with catalyzed hairpin assembly (CHA) signal amplification [31]. Triple-helix aptamer probes were formed involving an aptamer loop, two-segment stems, and a triplex oligonucleotide serving as the trigger probe. In the presence of tetracycline, the trigger probe was released from TAP, sparking the upcoming CHA amplification reaction in which two coexisting DNA hairpins (both modified with ferrocene) would hybridize into plentiful H1–H2 double helices. Afterward, Exonuclease III was added, demolishing double helices and simultaneously releasing ferrocene. The analytical signal was generated via the oxidation/reduction process of ferrocene, which was recognized by the β-cyclodextrin present on the gold electrode surface. The detection limit of this aptasensor is 0.13 nM. It reached a LOD comparable to that described in this work. However, the aptasensor proposed in this paper has superiority due to the simplicity of design and mechanism of action.

## 3. Materials and Methods

### 3.1. Reagents and Materials

Tetracycline hydrochloride (TET), oxytetracycline hydrochloride (OTC), doxycycline hydrochloride (DOX), 6-mercaptohexan-1-ol (MCH), sodium perchlorate (NaClO_4_), and sodium phosphate (Na_2_HPO_4_) were purchased from Sigma-Aldrich (Poznań, Poland). Potassium hydroxide, sulphuric acid, ethanol, and methanol were obtained from POCh (Gliwice, Poland). Alumina slurries with particle sizes of 0.3 µm and 0.05 µm were ordered from Buehler (Lake Bluff, IL, USA). The TET binding aptamer modified with ferrocene (Fc) HS-Apt-Fc:

5′–Fc–CCCCCGGCAGGCCACGGCTTGGGTTGGTCCCACTGCGCGT–(CH_2_)_3_–SH–3′, used as a probe, was synthesized by Biomers (GmbH, Germany). Commercially available UHT cow’s milk with 1.5% fat content was used as a natural matrix.

All electrochemical measurements and steps of aptasensor preparation were performed using a buffer containing 0.1 M NaClO_4_ & 2.5 mM Na_2_HPO_4_, pH 7.0.

All aqueous solutions were prepared with deionized and charcoal-treated water (resistivity of 18.2 MΩ/cm) purified with a Milli-Q reagent-grade water system (Millipore, Bedford, MA). All solutions were deoxygenated by purging with nitrogen (ultra-pure 6.0, Air Products, Warsaw, Poland) for 20 min.

### 3.2. Electrochemical Measurements

All electrochemical measurements, including cyclic voltammetry (CV) and square-wave voltammetry (SWV), were carried out on a potentiostat-galvanostat (Metrohm Autolab, The Netherlands) with a three-electrode configuration system. The gold disk electrodes with a 1.6 mm diameter were used as the working electrodes. Potentials were measured against the saturated-KCl silver chloride electrode (Ag/AgCl), and a platinum wire was used as the auxiliary electrode. All the electrodes used were purchased from Bioanalytical Systems (BASi, West Lafayette, IN, USA).

The electrode responses were expressed as the relative current changes:

(2) $$\Delta I=\frac{I_{n}-I_{0}}{I_{0}}\times 100\;\left[\%\right]$$

where *I_n_* is the peak current measured in the presence of the analyte’s particular concentration (n) and *I_0_* is the peak current registered in the buffer before the recognition process between the aptamer and antibiotic.

### 3.3. Fabrication of Electrochemical Aptasensors

Bare gold electrodes (Au) were first manually polished with 0.3 and 0.05 µm alumina slurry on a flat pad for 5 min each and carefully rinsed with Milli-Q water. Afterward, electrochemical cleaning of Au electrodes was carried out by CV. The method of electrochemical preparation of gold electrode surfaces comprises the following steps: Cleaning in 0.5 M KOH solution by cycling the potential between −400 and −120 0mV (vs. Ag/AgCl reference electrode) with a scan rate of 100 mV/s (3, 50, and 5 cycles) and in 0.5 M H_2_SO_4_ solution by sweeping the potential from −300 to +1500 mV at 100 mV/s (3, 10, and 5 scans). Ultimately, Au surfaces of the working electrodes were pre-treated via the application of ten CV cycles in a 0.5 M KOH solution. Upon completion of the electrochemical cleaning, each electrode was profusely rinsed with Milli-Q water and stored in water (for several minutes until the modification step). The applied gold electrode surface cleaning procedure was carried out according to the previously described protocol [23,33,34].

The modification of electrodes was performed according to the published protocol by Grabowska et al. [24]. Briefly, before modification, Au electrodes were rinsed with the buffer. Next, 10 μL of the solution comprised of 1 μM HS-Apt-Fc and 0.1 μM MCH in the buffer was dropped onto the surface of each electrode at room temperature and maintained for 3 h. In the second step of the modification, the electrodes were rinsed with the same buffer and then immersed in a 1 mM MCH buffer solution for 1 h. After rinsing with the same buffer, the electrodes were left overnight in the buffer at 4 °C until use.

#### 3.3.1. Cow Milk Sample Preparation

Before testing the actual sample, the cow milk was first diluted 1:10 with the buffer solution and then centrifuged at 15,000 rpm for 75 min at 20 °C to remove additional layers (fat and casein). After centrifugation, the cow milk samples separated into three layers: The top layer of fat, the middle layer of pure cow milk, and the bottom layer of casein. This procedure was adapted from the published protocol by Mohammad-Razdari et al. [35]. Fat and casein fractions were removed. Pure cow milk was filtered with a Millipore Amicon Ultracel YM-3, MWCO 3 kDa, and centrifuged for 75 min at 10,000 rpm at 20 °C in order to remove proteins with a molecular weight of over 3 kDa. Then, the TET standard solution was spiked into the cow milk matrix, making concentrations of 0.1, 0.5, and 1.0 nM. These solutions were used to carry out experiments for the detection of TET with the developed aptasensor.

#### 3.3.2. Electrochemical Measurements of Antibiotics

After overnight conditioning at 4 °C, the aptasensor was ready for electrochemical investigations. Ten microliters of a solution of variable tetracycline derivative (TET/OTC/DOX) concentrations (0.1, 0.5, 1.0, 5.0, and 10.0 nM) prepared in the buffer or in the pre-treated cow milk (see Section 3.3.1) was deposited onto the gold electrode surface modified with Apt-Fc. The electrodes were covered by Eppendorf tubes in order to protect against evaporation of the solution and air contamination. After 1h of interaction between Apt-Fc and antibiotic at room temperature, the electrodes were rinsed thoroughly with 5 mL of the buffer in order to remove the unbound targets. After that, electrodes were immersed in the same buffer, and square-wave voltammograms were recorded in the range of −100 to +800 mV with a 100 Hz frequency.

Each concentration of tetracyclines was measured separately using a single gold electrode modified with Apt-Fc. After the interaction with tetracyclines, Au-S-Apt-Fc/MCH electrodes were not regenerated for reuse.

## 4. Conclusions

In this study, we successfully developed an electrochemical aptasensor based on the assembly of HS-Apt-Fc and MCH for the detection of TET. The construction of this device was straightforward with great miniaturization potential. The specificity of the aptamer to TET and the sensitivity of SWV detection led to the sensitive and specific determination of TET in buffer and spiked cow milk samples with limits of detection of 0.16 nM (0.069 ng/mL) and 0.2 nM (0.088 ng/mL), respectively. The achieved LODs are significantly lower than the MRL limits of international regulations (225 nM, 1.0 × 10^4^ ng/mL, Commission Regulation 37/2010). Interfering antibiotics, such as OTC and DOX, have generated significantly weaker responses to the aptasensor, confirming its high selectivity. Thus, the aptasensor proved to be selective towards TET, with insignificant interferences both in the buffer and spiked cow milk samples.

In addition, the appropriate preparation of cow milk samples for analysis, which does not require complicated, long-duration procedures, causes a negligible influence of the matrix on the electrochemical measurements.

Moreover, this strategy has the advantage of being simple and allowing for low-cost mass production. The proposed device can provide an electrochemical platform for the future development of biosensors for other antibiotics in animal-origin milk and other food products.

## Figures and Tables

**Table 1:** Precision parameters characterizing the developed aptasensor based on Apt-Fc.

Repeatability					
Repetitions on the Same Electrode	1	2	3	4	5
i (µA)	0.75	0.78	0.77	0.77	0.79
Reproducibility					
Number of electrode	1	2	3	4	5
i (µA)	0.70	0.72	0.75	0.74	0.78
Stability					
Days	1		4	7	
i (µA)	0.75 ± 0.03		0.81 ± 0.04	0.80 ± 0.04	

**Table 2:** The values of relative intensity of current ΔI [%] measured with electrodes modified with the HS-Apt-Fc/MCH layer after incubation with TET, OTC, and DOX.

Concentration of TCs [nM]	TET		OTC		DOX		MIX ^b,c^
	Buffer ^a^	Spiked Cow Milk ^b^	Buffer ^a^	Spiked Cow Milk ^b^	Buffer ^a^	Spiked Cow milk ^b^	
0.1	28.7 ± 1.6	23.6 ± 2.6	−12.2 ± 2.0	2.8 ± 1.4	−2.2 ± 2.6	4.4 ± 1.0	19.8 ± 1.3
0.5	43.9 ± 2.5	44.6 ± 5.4	−18.5 ± 9.3		−6.9 ± 3.7		
1.0	67.9 ± 6.2	53.7 ± 3.5	25.2 ± 1.9		−1.8± 3.6		
5.0	93.4 ± 4.1		33.0 ± 7.6		2.9 ± 4.3		
10.0	99.8 ± 6.0		25.6 ± 4.3		6.7 ± 2.6		

Incubation conditions: ^a^ buffer containing 0.1 M NaClO_4_ & 2.5 mM Na_2_HPO_4_, pH 7.0; ^b^ pre-treated cow milk according to the procedure described in Section 3.3.1; ^c^ the mixed solution containing 0.1 nM TET, 0.1 nM OTC, and 0.1 nM DOX.

**Table 3:** Comparison of different methods for tetracycline detection in cow milk samples.

Method	Detection Limit (ng/mL)	Reference
Bacillus cereus test system	30	[29]
Bacillus calidolactis tube diffusion test	45	[29]
Charm HVS receptor assay	150	[29]
HPLC	10	[29]
Test-lux microbiological test	6.3	[30]
Snap immunoassay	6.3	[30]
Delvotest SP microbial inhibition test	500	[30]
Electrochemical platform based on ssDNA aptamer labelled with ferrocene	0.088	This work

## Data Availability

The data presented in this study are available on request from the corresponding author.
